# Dr. Parks’s Article

**Published:** 1900-10

**Authors:** 


### DR. PARKS'S ARTICLE.

WE have in this issue a common sense and honest exposition of Therapeutic Psychology by Dr. W. B. Parks of Atlanta. The subject is reviewed as understood by a conscientious physician, and the article is worthy of careful attention.

---

DEATH has been busy with the distinguished medical men. During the last few weeks J. M. Da Costa and Alfred Stillé, Philadelphia, have been taken, thus depriving physical diagnosis and therapeutics of two shining lights. Lewis A. Sayre, the veteran orthopedist, was not long behind Hunter McGuire. The places that these men occupied will be hard to fill.

